# Embryogenesis and tadpole description of *Hyperolius castaneus* Ahl, 1931 and *H. jackie* Dehling, 2012 (Anura, Hyperoliidae) from montane bog pools

**DOI:** 10.3897/zookeys.546.6044

**Published:** 2015-12-16

**Authors:** Edgar Lehr, J. Maximilian Dehling, Eli Greenbaum, Ulrich Sinsch

**Affiliations:** 1 Department of Biology, Illinois Wesleyan University, P.O. Box 2900, Bloomington, IL 61701, USA; 2 Institute of Integrated Sciences, Department of Biology, University of Koblenz–Landau, Universitätsstr. 1, D–56070 Koblenz, Germany; 3 Department of Biological Sciences, University of Texas at El Paso, 500 West University Avenue, El Paso, TX 79968, USA

**Keywords:** Cryptic species diversity, egg predation, egg laying behavior, frogfly, Nyungwe National Park, Rwanda

## Abstract

Tadpoles of *Hyperolius castaneus* and *Hyperolius jackie* were found in the Nyungwe National Park in Rwanda and adjacent areas. Tadpoles of both species were identified by DNA-barcoding. At the shore of a bog pool three clutches of *Hyperolius castaneus* of apparently different age, all laid on moss pads (*Polytrichum commune*, *Isotachis aubertii*) or grass tussocks (*Andropogon shirensis*) 2–5 cm above the water level, were found. One clutch of *Hyperolius castaneus* was infested by larval dipterid flies. The most recently laid clutch contained about 20 eggs within a broad egg-jelly envelope. The eggs were attached to single blades of a tussock and distributed over a vertical distance of 8 cm. A pair of *Hyperolius castaneus* found in axillary amplexus was transported in a plastic container to the lab for observation. The pair deposited a total of 57 eggs (15 eggs attached to the upper wall of the transport container, 42 eggs floated in the water). Embryogenesis of the clutch was monitored in the plastic container at 20 ± 2 °C (air temperature) and documented by photos until Gosner Stage 25. The description of the tadpole of *Hyperolius castaneus* is based on a Gosner Stage 29 individual from a series of 57 tadpoles (Gosner stages 25–41). The description of the tadpole of *Hyperolius jackie* is based on a Gosner Stage 32 individual from a series of 43 tadpoles (Gosner stages 25–41). Egg laying behavior and embryogenesis are unknown for *Hyperolius jackie*. The labial tooth row formula for both species is 1/3(1) with a narrow median gap of the tooth row. Variation in external morphology was observed in size and labial tooth row formula within the species. With the tadpole descriptions of *Hyperolius castaneus* and *Hyperolius jackie*, 36 tadpoles of the 135 known *Hyperolius* species have been described, including five of the eleven *Hyperolius* species known from Rwanda.

## Introduction

The reed frog genus *Hyperolius* currently comprises 135 species (Frost 2015). Taxonomy of this genus is known to be complicated (e.g., Ahl 1931, Schiøtz 1975, 1999, Lötters et al. 2004, Rödel et al. 2010) because of high intraspecific variability, high interspecific morphological similarity, and sympatric distributions (e.g., Channing et al. 2013, Liedtke et al. 2014). Not surprisingly, the tadpoles of only 34 (24.8%) *Hyperolius* species have been described to date (Viertel et al. 2007, Channing et al. 2012, Conradie et al. 2013), a serious drawback for a reliable assessment of the presence of species in remote regions where adults are not easily caught (e.g. Greenbaum et al. 2013).

During our recent field work in Rwanda, we focussed on the estimation of *Hyperolius* diversity, specifically in the Nyungwe National Park (about 970 km² cloud forest, Plumptre et al. 2003; for a map see Dehling 2012: page 60, figure 4). Despite a century of taxonomic studies (Ahl 1931, Hinkel and Fischer 1990, 1995, Fischer and Hinkel 1992, Hinkel 1996, Sinsch et al. 2011, Dehling 2012) diversity of the cloud forest *Hyperolius* from that area is not yet clear. The checklist of Hinkel (1996) mentions *Hyperolius adolfifriederici* Ahl, 1931, *Hyperolius alticola* Ahl, 1931, *Hyperolius castaneus* Ahl, 1931, *Hyperolius discodactylus* Ahl, 1931, *Hyperolius raveni* Ahl, 1931 and *Hyperolius viridiflavus francoisi* Laurent, 1951, several of which are now considered junior synonyms (Frost 2015). Our current view integrating morphological, bioacoustics and molecular data gives credit to the presence of only four species in the Nyungwe National Park: *Hyperolius castaneus*, *Hyperolius discodactylus*, *Hyperolius frontalis* Laurent, 1950 and the recently described *Hyperolius jackie* Dehling, 2012 (Sinsch et al. 2011, Dehling 2012, Greenbaum et al. 2013, Liedtke et al. 2014, Dehling unpubl. data). Analysing habitat preferences and distribution of these four species within the cloud forest and the adjacent areas now deforested and in agricultural use would be easier, if encountered tadpoles could be assigned to either taxon. Yet, none of the tadpoles are currently described (Channing et al. 2012). Consequently, we surveyed lentic water bodies for *Hyperolius* tadpoles of these four species at all localities where we previously detected the presence of either species by collection of specimens or based on advertisement calls (Sinsch et al. 2011, Dehling 2012, Greenbaum et al. 2013, Liedtke et al. 2014). This survey yielded a large number of tadpoles which we identified as those of *Hyperolius castaneus* and *Hyperolius jackie* by DNA-barcoding. Herein we describe the morphological features of the tadpoles and provide new information on the egg-laying behavior of *Hyperolius castaneus* and embryogenesis in their terrestrial clutches.

## Methods

### Study areas and field surveys

Presence of larval and adult individuals of *Hyperolius castaneus* and *Hyperolius jackie* was monitored in the Nyungwe National Park, Rwanda (Sinsch et al. 2011, Dehling 2012) and adjacent areas used for agriculture (Table 1). Daytime surveys (9.00–17.00) for tadpoles and nightly records (18.00–21.00) of calling males were conducted in March 2009, March and April 2011 and in March 2012. *Hyperolius castaneus* egg laying behavior was studied in the Uwasenkoko swamp. Tadpoles of *Hyperolius castaneus* were collected at the same site and additionally in the Karamba swamp together with those of *Hyperolius jackie* (Table 1). Additional tadpole specimens were collected from multiple localities in the Albertine Rift in Democratic Republic of Congo and Uganda. Museum acronyms are: UTEP = University of Texas at El Paso, ZFMK = Zoologisches Forschungsmuseum Alexander Koenig, Bonn (Appendix I).

**Table 1.** Localities where *Hyperolius castaneus* and *Hyperolius jackie* adults (= A) and tadpoles (= T) were collected in Rwanda.

Locality	Latitude [°S], Longitude [°E]	Altitude [m a.s.l.]	*Hyperolius castaneus*		*Hyperolius jackie*	
			A	T	A	T
Gisakura	2.457, 29.092	1927	+	-	-	-
Kitabi	2.546, 29.426	2190	+	-	-	-
Nyungwe, stream	2.464, 29.101	1881	+	-	+	-
Nyungwe, Kamiranzovu	2.486, 29.153	1961	+	-	-	-
Nyungwe, Karamba	2.479, 29.112	1936	+	+	+	+
Nyungwe, Pindura	2.481, 29.228	2283	+	-	-	-
Nyungwe, Uwasenkoko	2.529, 29.354	2379	+	+	-	-

### Larval characters

The format of the tadpole description follows that of Viertel et al. (2007) but excludes description of oral cavities. Tadpoles were preserved in 5–10% formalin. Body measurements follow the primary landmarks defined by McDiarmid and Altig (1999: see figure 3.1 on page 26 for tadpole drawing with defined primary landmarks). In our descriptions, we use the terminology of Altig (1970) and McDiarmid and Altig (1999) with the labial tooth row formula (LTFR) written as a fraction in line with the rows with median gaps in parentheses. P1 = first posterior tooth row. Ecomorphological types for larvae follow McDiarmid and Altig (1999) and Orton (1953). Tadpoles were staged according to Gosner (1960). Preserved tadpoles were observed on tiny glass beads (1 mm) filled shallowly with water to allow proper positioning. Most measurements were taken to the nearest 0.1 mm using a stereomicroscope equipped with an ocular micrometer, except for tail length, body length, body width, and greatest tail height, which were measured with a digital caliper held under the microscope.

Recorded measurements include: body length (distance from the tip of the snout to the body terminus, which is the junction of the posterior body wall with the tail axis); tail length (distance from the body terminus to the absolute tip of tail); total length (sum of body length and tail length); body width (measured at the widest point right behind the eyes); body height (at level of eye); eye diameter; interorbital distance (measured between the centers of the pupils); internarial distance (measured between the centers of the nostril indicated by reduced pigmentation when closed); distance between tip of snout and naris (from center of the naris to the middle of the snout); and distance between nostril and eye (from the center of nostril to the anterior edge of the eye); spiracle length (medially to opening); and spiracle tube width (at level of opening), and oral disc width (at middle between outer marginal papillae). Drawings of tadpoles were done with a camera lucida attached to a microscope. Descriptions of coloration in life are based on photos taken by JMD shortly after collection in the field.

### DNA sampling and barcoding

We isolated DNA from the tail tip of the tadpole morphotypes, collected at the Karamba and Uwasenkoko localities (Table 1). DNA was used to sequence a fragment of the 16S mitochondrial rRNA gene, a suggested universal marker to barcode amphibians for species allocation (Vences et al. 2005). Protocols of DNA extraction, PCR, purification, and sequencing follow Dehling and Sinsch (2013) and Greenbaum et al. (2013). The obtained sequences were compared with our own sequences from adult frog specimens collected in southwestern Rwanda and are deposited in GenBank (Table 2). Editing and alignment were completed in MEGA5 (Tamura et al. 2011). Sequences were trimmed to the same length. The final alignment consisted of 548 base pairs. Calculations of pairwise distances and phylogenetic analysis (Maximum Likelihood) were carried out in MEGA5. A Maximum Likelihood analysis was run with 1000 bootstrap replicates using the GTR + G + I model and the Nearest-Neighbor-Interchange, as proposed by jModelTest 2 (Darriba et al. 2012) using the Akaike information criterion.

**Table 2.** Samples of species used for molecular genetic analyses, their geographic origin, voucher specimens (T = tadpole, otherwise adult), GenBank accession numbers, and original source.

Species	Origin	Voucher	GenBank #	Source
*Afrixalus quadrivittatus*	Butare	JMD544	KT439195	This study
*Hyperolius castaneus*	Nyungwe National Park	ZMB 77537	JQ423936	Dehling 2012
*Hyperolius castaneus*	Uwasenkoko, Nyungwe National Park	ZFMK 97191, T	KT439194	This study
*Hyperolius castaneus*	Karamba	ZFMK 97192, T	KT439193	This study
*Hyperolius cinnamomeoventris*	Butare	ZMB 77533	JQ966568	Dehling 2012
*Hyperolius discodactylus*	Nyungwe National Park Rwanda	ZMB 77536	JQ966565	Dehling 2012
*Hyperolius jackie*	Karamba, Nyungwe National Park	ZMB 77481	JQ966571	Dehling 2012
*Hyperolius jackie*	Karamba, Nyungwe National Park	ZFMK 97194, T	KT439192	This study
*Hyperolius kivuensis*	Butare	ZMB 77532	JQ966567	Dehling 2012
*Hyperolius lateralis*	Butare	ZMB 77534	JQ966569	Dehling 2012
*Hyperolius rwandae*	Akagera wetland	ZMB 77225	JQ863713	Channing et al. 2013
*Hyperolius rwandae*	Butare	JMD 592	KT439191	This study
*Hyperolius viridiflavus*	Gitarama	ZMB 77535	JQ966570	Dehling 2012
*Leptopelis karissimbensis*	Uwasenkoko swamp, Nyungwe National Park	ZFMK 97188, T	KT439190	This study
*Leptopelis karissimbensis*	Uwasenkoko swamp, Nyungwe National Park	JMD 631	KT439189	This study
Leptopelis cf. kivuensis 2	Karamba, Nyungwe National Park	ZFMK 97189, T	KT439188	This study
Leptopelis cf. kivuensis 2	Karamba, Nyungwe National Pak	JMD 746	KM047142	Portillo et al. 2015

## Results

### Distribution and habitat preferences of *Hyperolius* spp. in the Nyungwe region

**Figure 1.**
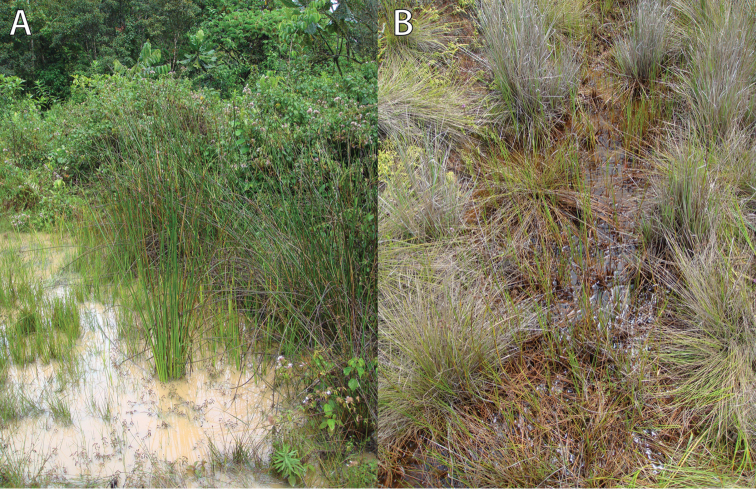
Tadpole habitats in the Nyungwe National Park. **A** Karamba swamp **B** Uwasenkoko swamp. For geographical details see Table 1. Photos by U. Sinsch.

Based on call surveys and collection of adult specimens, *Hyperolius castaneus* populations were detected at seven localities, five inside the Nyungwe National Park, and two outside (Table 1). They occured in sympatry with *Hyperolius discodactylus*, *Hyperolius jackie*, *Leptopelis karissimbensis* Ahl, 1929, Leptopelis cf. kivuensis 2 (sensu Portillo et al. 2015), *Phrynobatrachus acutirostris* Nieden 1912, “1913”, Phrynobatrachus cf. versicolor Ahl, 1924, *Xenopus wittei* Tinsley, Kobel & Fischberg, 1979 and an undetermined species of *Amietia* Dubois, 1987 “1986”. *Hyperolius castaneus* tadpoles shared the same lentic water bodies with those of *Hyperolius jackie*, *Leptopelis karissimbensis* and Leptopelis cf. kivuensis 2 (Fig. 1). *Hyperolius jackie* populations are currently known only from the type locality (a natural pond at Karamba, Nyungwe National Park), and a stream at the west end of the Nyungwe National Park (Table 1). Adults were found in sympatry with *Hyperolius castaneus*, *Hyperolius discodactylus*, *Leptopelis karissimbensis* and *Xenopus wittei*; and tadpoles syntopically with those of *Hyperolius castaneus* and Leptopelis cf. kivuensis 2. *Hyperolius discodactylus* tadpoles were found syntopically with tadpoles of *Phrynobatrachus acutirostris* in a slow flowing stream passing through the Uwasenkoko swamp.

Males of *Hyperolius castaneus* and *Hyperolius jackie* were observed vocalizing from shrubs and sedges bordering forest swamps. *Hyperolius castaneus* also called from the ground in moist swamp areas. While *Hyperolius jackie* never started vocalizing before dusk, *Hyperolius castaneus* gave advertisement calls throughout the day, but more frequently at night. Bog pools close to calling sites and containing tadpoles had a pH of 5.5–6.0 and a water depth varying from a few centimetres to a maximum of 35 cm (Fig. 1).

### Egg-laying behavior and embryogenesis of *Hyperolius castaneus*

**Figure 2.**
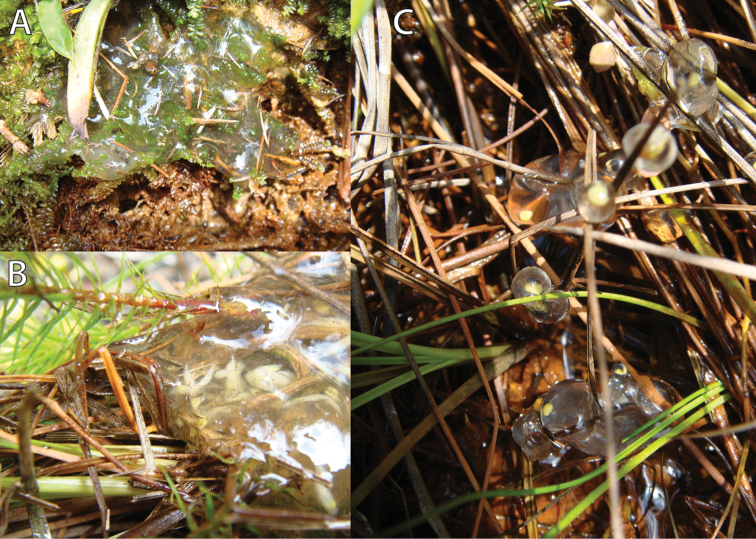
*Hyperolius castaneus* clutches of different age at the Uwasenkoko swamp. **A** Gelatinous clutch mass following hatching of tadpoles **B** Parasitized clutch mass with a few undeveloped eggs **C** Recently laid eggs attached to rush stalks. For further details see text. Photos by U. Sinsch.

The natural history observations reported here were made on 22 March 2012 between 13:00 and 16:00 hrs, at a small breeding pond forming part of the Uwasenkoko swamp (2379 m a.s.l.; Fig. 1B). During an initial survey of a 25 m² area, we located two males advertising at the ground and an unpaired female, all individuals staying 3–8 m apart from each other. At the shore of the pond we detected three clutches of different ages, laid on moss pads and grass tussocks 2–5 cm above the water level (Fig. 2). The first clutch mass was placed on a moss pad (*Polytrichum commune*, *Isotachis aubertii*) and consisted only of the gelatinous remains of the egg envelopes (Fig. 2A). According to the duration of embryogenesis (see below) we estimate the age of this clutch is at least seven days. The second clutch was found upon depressed blades of mainly *Andropogon shirensis* (Fig. 2B) and had a similar consistency to the first one. However, with the exception of three undeveloped eggs, it contained a large number of undetermined insect larvae, probably of parasitic dipterid flies. The third clutch was recently laid with about 20 eggs within the broad egg-jelly envelope. The eggs were attached to single blades of an *Andropogon shirensis* tussock and distributed over a vertical distance of 8 cm (Fig. 2C). The eggs had a black pole cap, whereas about two-thirds of the egg was yellowish. Within the shallow water adjacent to the clutches we observed > 50 *Hyperolius castaneus* tadpoles (Gosner stages 25–31, one metamorphic individual with lateral yellowish stripes of Stage 41) and > 15 *Leptopelis karissimbensis* tadpoles (Gosner stages 35–39). The developmental stage of most tadpoles indicated that they had hatched recently. We conclude that a reproductive burst of several pairs had occurred 1–2 weeks prior to the survey, but that reproduction period is prolonged with little synchronisation among the several hundred local *Hyperolius castaneus* adults.

**Figure 3.**
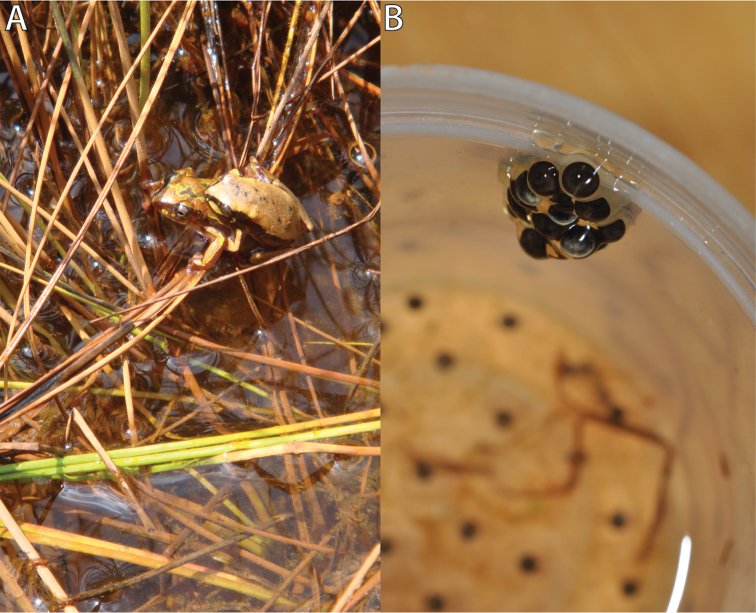
**A** *Hyperolius castaneus* pair in amplexus at the Uwasenkoko swamp **B** Clutch laid in the transport box; 15 eggs attached to the upper container wall and 42 eggs within the water. For further details see text. Photos by U. Sinsch and M. Dehling.

During the same survey we observed a pair in axillary amplexus on shore close to the open water surface (Fig. 3A). The male did not call and during the next two hours the pair moved occasionally along the shoreline. As the pair did not oviposit during this period, they were transferred into a small plastic container (5 cm diameter, 12 cm height, containing water to a height of 4 cm) and transported to the laboratory in Butare at 1643 m a.s.l. Reaching the laboratory two hours later we found that the pair had laid 15 eggs attached to the upper wall of the transport container and another 42 eggs were floating in the water (Fig. 3B). Eggs were deposited one by one using the egg-jelly envelope as glue for attachment to the wall and among single eggs. The pair, which already had finished amplexus, was removed from the box. Embryogenesis of the clutch was monitored in the same transport container at 20 ± 2 °C, but at a significantly higher air temperature compared to the native Uwasenkoko locality where daily fluctuations between 5 and 19 °C occur.

**Figure 4.**
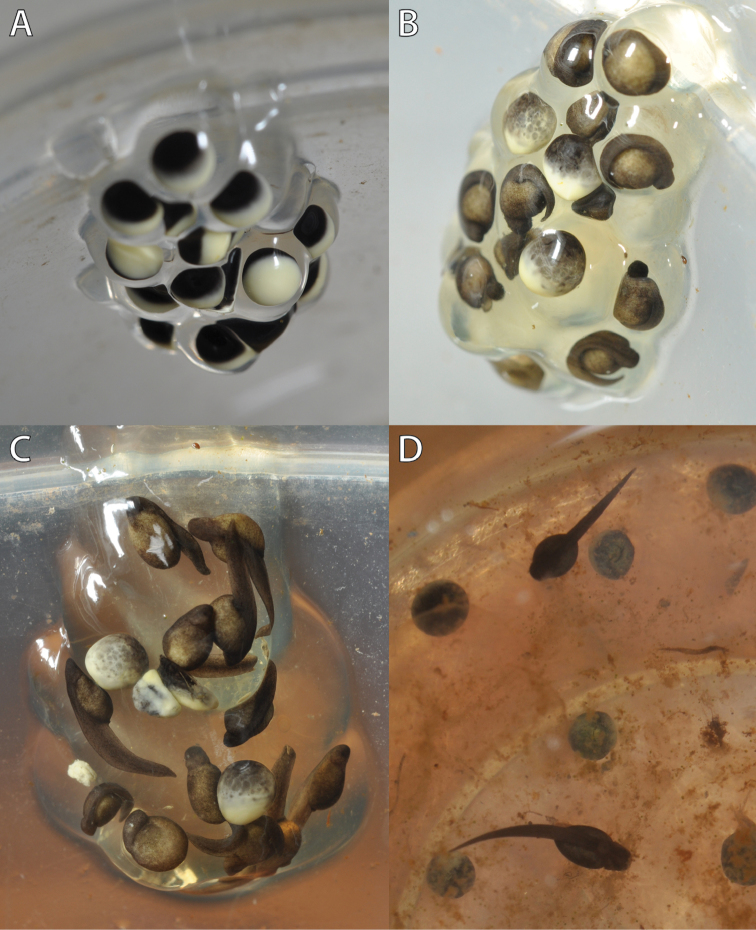
Embryogenesis of a *Hyperolius castaneus* clutch at 20 ± 2 °C. **A** Egg mass 4 cm above water level 6h following oviposition **B** 5d following oviposition **C** 6d following oviposition **D** 7d following oviposition; two hatchlings of the upper egg mass and undeveloped eggs within water. For further details see text. Photos by M. Dehling.

**Figure 5.**
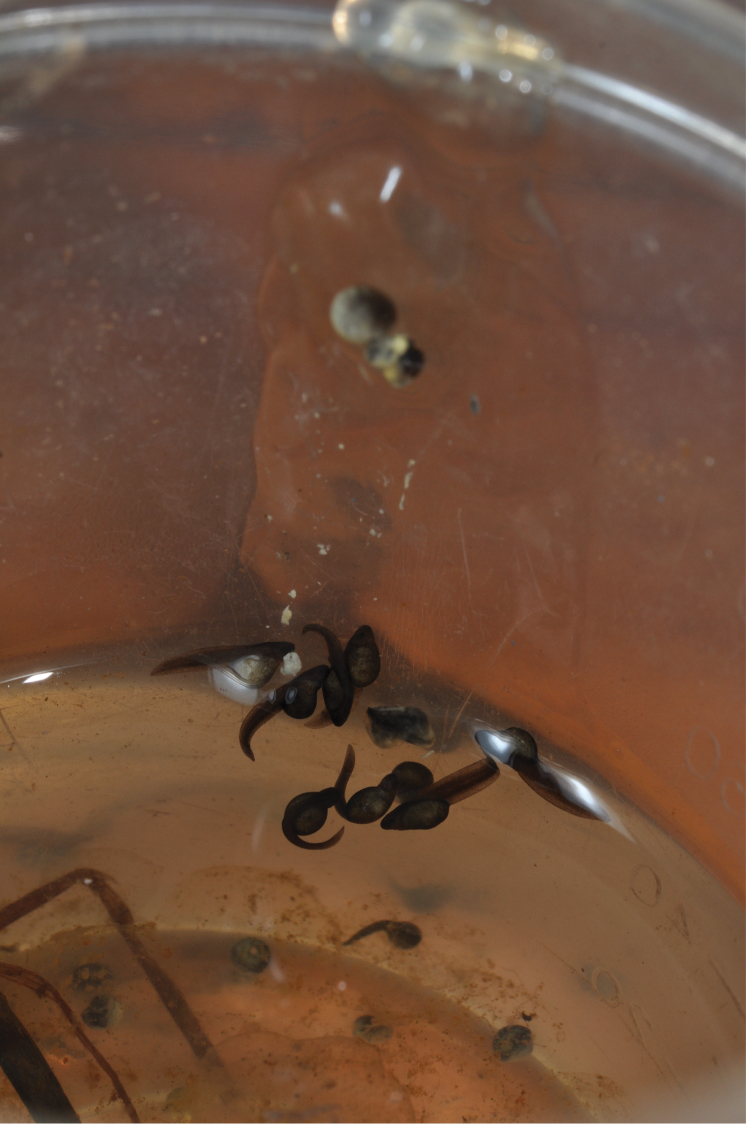
Hatching of *Hyperolius castaneus* tadpoles from an egg mass attached 4 cm above water level. For further details see text. Photos by M. Dehling.

Six hours after oviposition the first eggs of the upper egg mass showed signs of cleavage (Gosner Stage 2; Fig. 4A). The egg envelope was not swollen by moisture uptake, but each single egg remained distinguishable. After 48 h most eggs were in a stage of gastrulation (Gosner stages 10–13). After 5 d the most advanced embryos had reached Gosner Stage 19 (Fig. 4B), and after 6 d embryos reached Gosner Stage 22 and egg envelopes had fused to a single swollen gelatinous mass (Fig. 4C). Between 6 and 7 d following oviposition the egg-jelly became more fluid and the late embryos and early tadpoles of Gosner stage 24–25 started moving within the egg mass. At the end of day 7 the most advanced tadpoles had moved downwards within the egg-jelly, reaching the water level and beginning their free-swimming tadpole stage (Figs 4D, 5). In general, embryonic development of the 15 eggs was slightly asynchronic and two eggs did not seem to be fertilized (Fig. 5). In contrast, eggs deposited in water failed to develop further than Gosner Stage 10.

### DNA-barcoding of tadpoles

**Figure 6.**
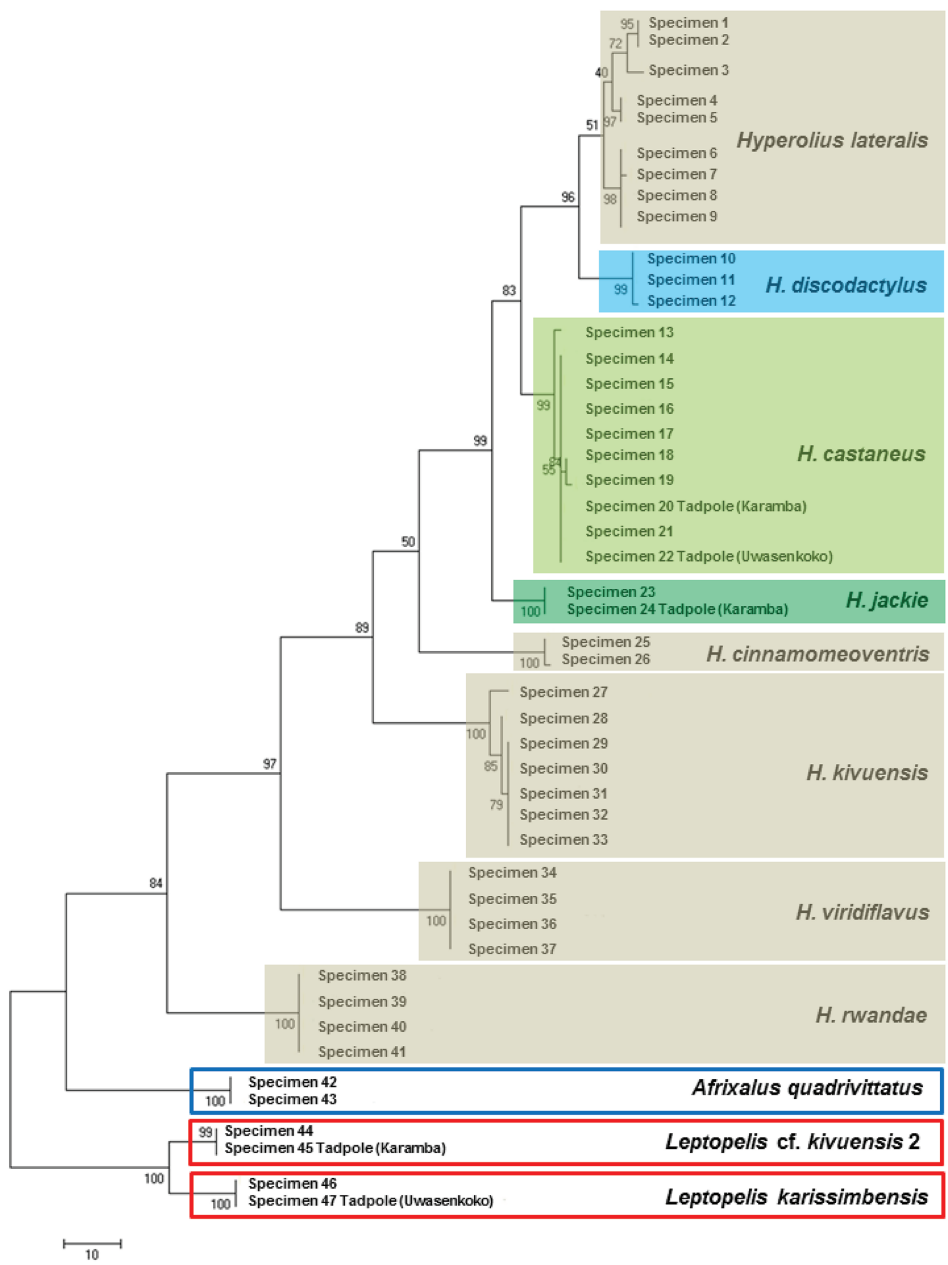
Maximum likelihood phylogram of Rwandan species in the genus *Hyperolius* with *Afrixalus quadivittatus*, *Leptopelis karissimbensis* and Leptopelis cf. kivuensis 2 as outgroups, based on comparison of 548 base pairs of the mitochondrial 16S rRNA gene. Included are 42 adult specimens collected in southwestern Rwanda, samples taken from GenBank and five tadpoles representing the morphotypes collected in the Karamba and Uwasenkoko swamps (specimen identification in Appendix I). Numbers above nodes are percentage support values from maximum likelihood. Only values above 50% are shown.

DNA-sequences of representative specimens of the three morphologically distinct tadpole types collected in the Karamba pond and of the two tadpole types collected in the Uwasenkoko swamp were unequivocally associated (uncorrected p distance 0.0% between tadpole and corresponding adult sequence) with adult sequences of *Hyperolius castaneus*, *Hyperolius jackie*, *Leptopelis karissimbensis*, and Leptopelis cf. kivuensis 2 (Fig. 6).

### Tadpole of *Hyperolius castaneus* Ahl, 1931

**Figure 7.**
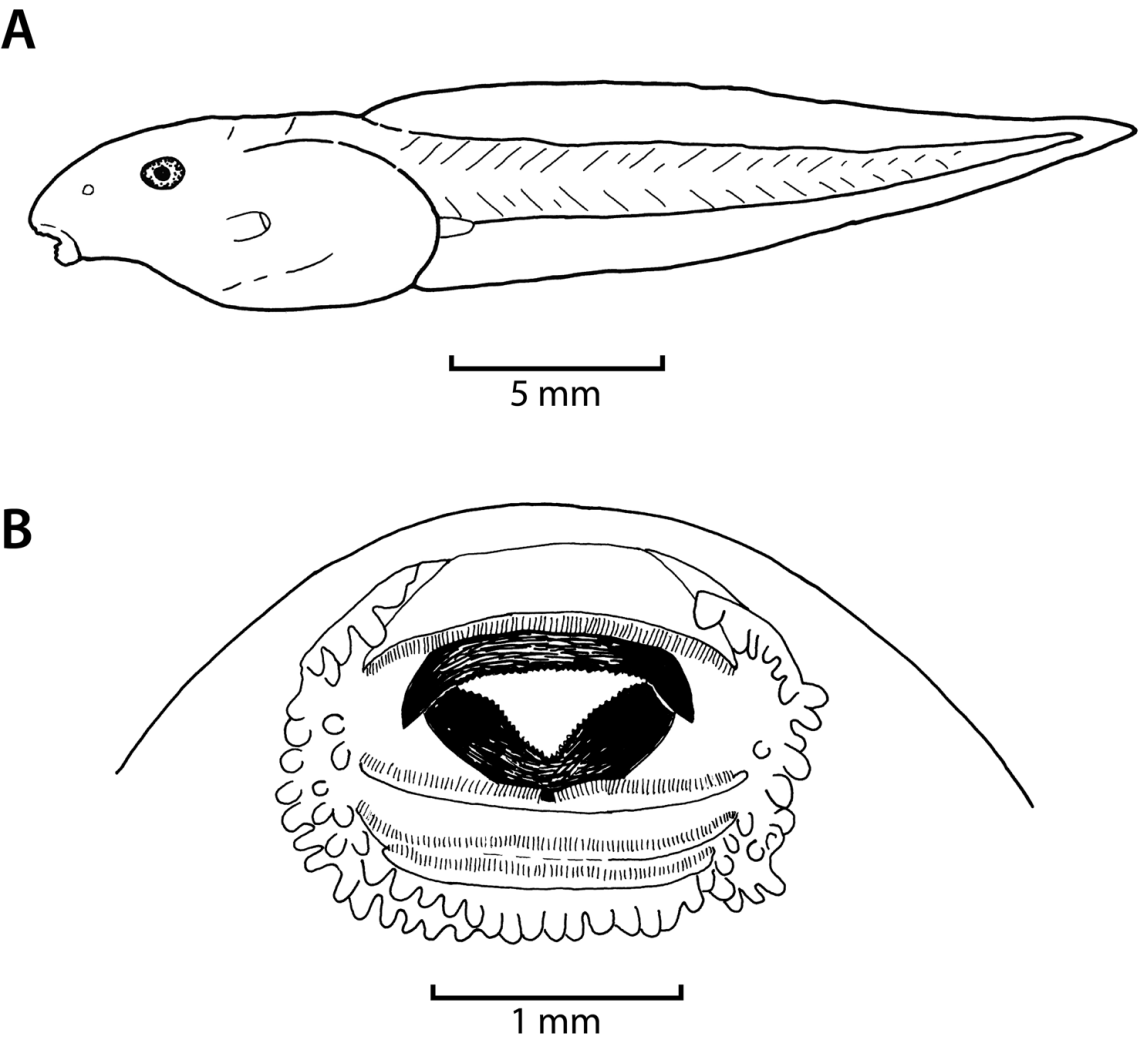
Tadpole of *Hyperolius castaneus* (Stage 29, ZFMK 97190) in lateral view (**A**) and oral disc (**B**). Drawings by E. Lehr.

**Figure 8.**
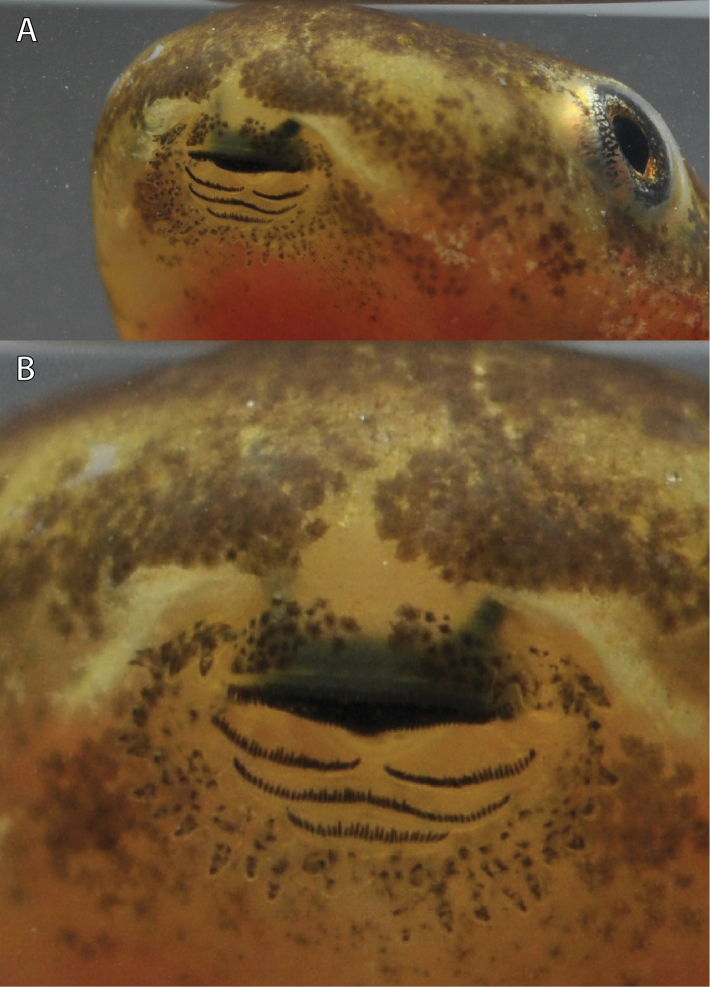
Oral disc in life of *Hyperolius castaneus* (Gosner Stage 40, from Karamba, ZFMK 97192) in overview (**A**) and close up view (**B**). Photos by M. Dehling.

**Figure 9.**
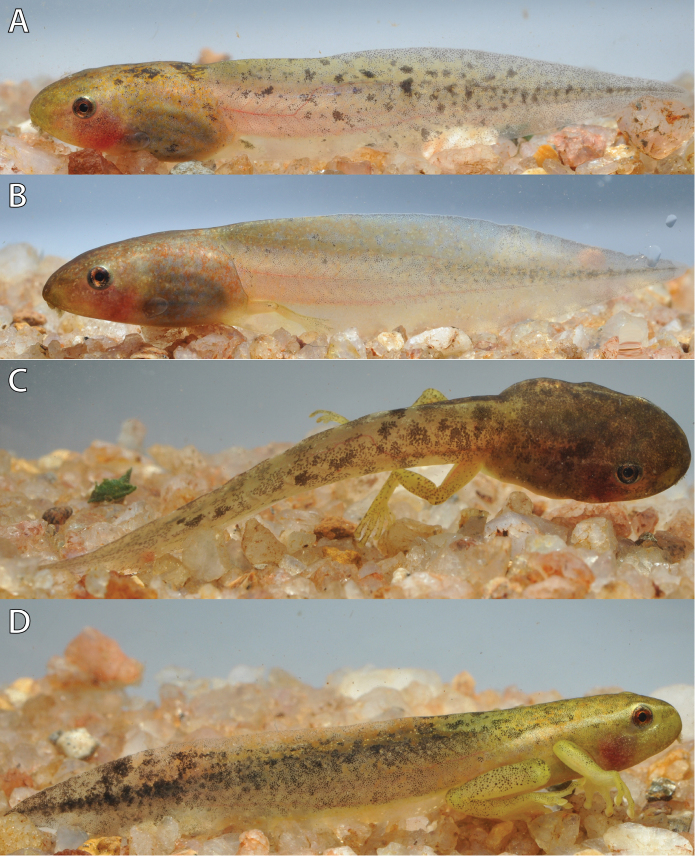
Color variation in life of *Hyperolius castaneus* from Karamba (ZFMK 97192) at different Gosner stages. **A** Stage 35 **B** Stage 37 **C** Stage 38 **D** Stage 44. Photos by M. Dehling.

**Figure 10.**
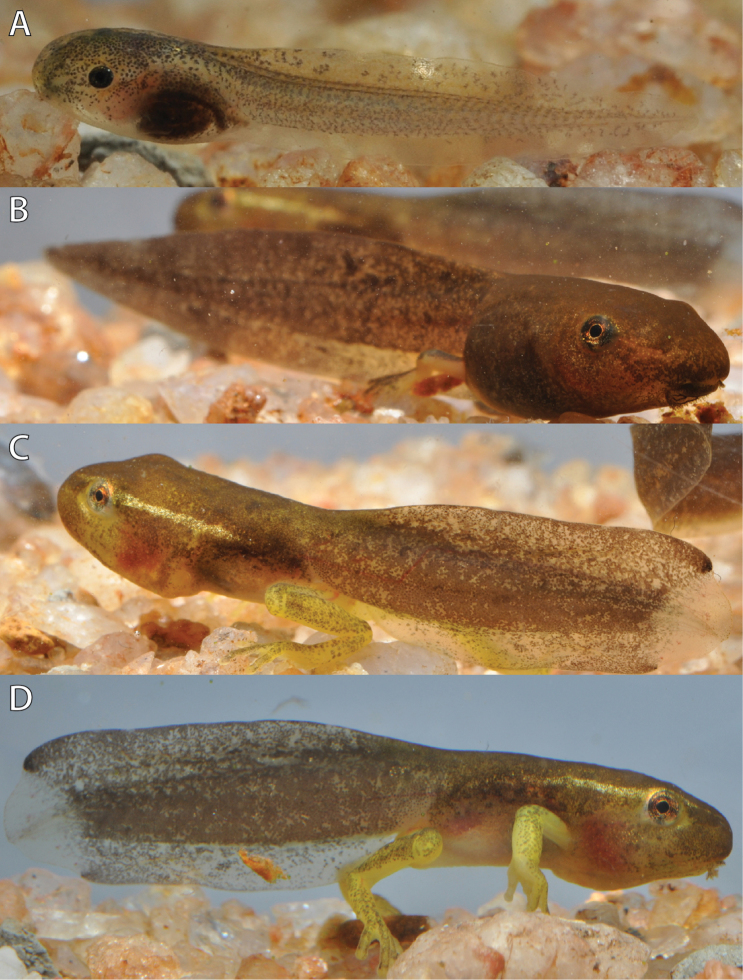
Color variation in life of *Hyperolius castaneus* from Uwasenkoko (ZFMK 97191) at different Gosner stages. **A** Stage 25 **B** Stage 38 **C** Stage 41 **D** Stage 44. Photos by M. Dehling.

The following description is based on a Stage 29 individual from the Uwasenkoko swamp, Rwanda (Figs 7A, B, ZFMK 97190, selected from a series of 52 tadpoles, Gosner stages 25–38, ZFMK 97191, and a series of 5 tadpoles, Gosner stages 34–41, ZMFK 97192 from Karamba, Figs 8–10). Exotrophous lentic benthic Type IV tadpole with following measurements (mm): total length 24.0, body length 9.0, tail length 15.0, body width 4.7, body height 3.6, eye diameter 1.0, interorbital distance 4.0, internarial distance 2.7, snout–naris–distance 1.9, distance–naris–eye 1.6, spiracle length 1.7, spiracle width 1.0, distance–snout–spiracle 6.4, tail muscle height at its beginning 2.4, tail muscle height at tail mid-length 1.8, greatest tail height 4.0, oral disc width 2.3. In dorsal view the body is elongated and ovoid and is widest at the level of the spiracle opening. The snout is rounded both in lateral and dorsal views. The interorbital distance is about twice the snout–naris distance, and internarial distance is 68% of interorbital distance. The eyes are positioned laterally, directed dorsolaterally, and are not visible in ventral view. The external nares are nearly round (slightly elongated horizontally), very small, and positioned laterally. They are more closely positioned to the eyes than to the snout (naris–eye–distance to snout–naris–distance 84%). In lateral view the body is highest at the mid-body length (approximately at the level of the spiracle opening). The body height is 40% of the body length, the body width is about half (52%) the length of the body, and the body height is 77% of the body width. The spiracle is single, sinistral, and attached to the body wall. Its shape is cylindrical and its length is about twice (170%) the eye diameter. The spiracle opening is rounded, directed posteriorly, and located at mid-body with its upper margin below the lower margin of the eye in lateral view. The length of the tail represents 63% of the total length. The tail is highest at about mid-tail and represents about a quarter (27%) of the tail length. The greatest tail height is located at the anterior quarter of the tail. The greatest tail height is slightly more than twice (225%) the body length, and slightly larger (111%) than the body height. The dorsal fin does not extend onto the body. Dorsal and ventral fins are about equal in height throughout their length. The tip of the tail is narrowly pointed and rounded. The height of the tail musculature at mid-body is about half (45%) of the maximum tail height. The vent tube is dextral, short, posteriorly directed, and linked to the tail musculature. The oral disc (Figs 7B, 8) is anteroventral, not emarginated, about half (49%) of the body width, and bordered at its lateral and posterior margin by a row of short and round papillae. Few submarginal papillae are present laterally and below the third lower tooth row. The LTRF is 1/3(1) with a narrow median gap in P1. The first two tooth rows are about equal in length, occupying nearly the entire width of the oral disc, the third tooth row is slightly shorter, and the shortest is the most posterior one. Jaw sheaths are finely serrated. The upper jaw sheath is inversely U-shaped and the lower V-shaped and narrower.

The variation in external morphology of the larval series is limited to size (Table 3) and LTRF. Fourteen tadpoles differ from the above described LTRF: Seven tadpoles had a LTRF of 1/3(1, 3), three of 1/3(1, 2), two of 1/3(1, 2, 3), one of 1(1)/3, and one of 1/3. *Hyperolius castaneus* tadpoles from outside Rwanda (see Appendix I) correspond well with the description. One tadpole from Uganda (UTEP 21179) had a LTRF of 1(1)/3(1).

**Table 3.** Measurements (mm) of 57 larvae of *Hyperolius castaneus*. Mean followed by one standard deviation, and range in parentheses for sample sizes larger than 2.

*Hyperolius castaneus*				
Stage	N	Total length	Body length	Tail length
**25**	14	11.3–16.0 (13.4 ± 1.4)	3.7–5.1 (4.5 ± 0.4)	7.6–10.9 (9.0 ± 1.0)
**26**	6	18.6–20.7 (20.2 ± 0.8)	6.5–7.2 (7.0 ± 0.3)	12.1–13.5 (13.2 ± 0.5)
**27**	4	19.4–25.5 (22.3 ± 2.5)	7.2–8.6 (7.8 ± 0.6)	12.2–16.9 (14.5 ± 1.9)
**28**	2	23.7, 23.9	8.5, 8.6	15.2, 15.3
**29**	2	24.0, 27.2	9.0, 9.6	15.0, 17.6
**31**	4	25.0–28.9 (25.7 ± 3.0)	9.1–9.3 (9.2 ± 0.1)	12.7–19.6 (16.5 ± 2.9)
**34**	5	27.8–32.7 (29.6 ± 2.0)	9.4–11.0 (10.1 ± 0.6)	18.4–20.3 (19.6 ± 1.4)
**35**	6	29.3–33.0 (31.1 ± 1.5)	10.0–10.9 (10.6 ± 0.4)	19.1–22.0 (20.6 ± 1.3)
**36**	5	30.8–33.0 (31.9 ± 0.9)	9.7–11.7 (10.8 ± 0.7)	20.1–23.0 (21.2 ± 1.2)
**37**	4	32.9–34.9 (33.9 ± 0.8)	10.0–11.5 (11.1 ± 0.7)	22.3–22.9 (22.8 ± 0.5)
**38**	2	32.1, 33.1	10.6, 11.1	21.5, 22.0
**39**	2	33.3, 34.2	10.5, 10.6	22.8, 23.6
**41**	1	31.0	10.7	20.3

In preservative the larvae are entirely pale grayish brown to tan. The body is darker dorsally compared to the translucent venter. Tail musculature is tan and the fins are translucent, both bearing dark gray melanophores in various degrees.

The coloration in life (Figs 9, 10) of the body was dorsally tan with minute brownish-orange spots and translucent whitish on the venter. The tail musculature was greenish tan and the fins were translucent tan with irregular dark marbling. Black spots and flecks were scattered dorsally and laterally on the body, tail musculature and dorsal fin. The ventral fin has fewer black spots and flecks or none at all. Younger stages (e.g., Gosner Stage 25, Fig. 10A) are paler compared to older stages (e.g., Gosner Stage 38, Fig. 10B). The series from Uwasenkoko was overall darker (e.g., Gosner Stage 38, Fig. 10B) compared to the series from Karamba (e.g., Gosner Stage 37, Fig. 9), possibly reflecting phenotypic plasticity. From stages 38 on in both series, distinct tan or whitish yellow dorsolateral stripes are present on each side extending from the snout to the end of the body. The iris was brownish orange with a few dark gray reticulations.

### Tadpole of *Hyperolius jackie* Dehling, 2012

**Figure 11.**
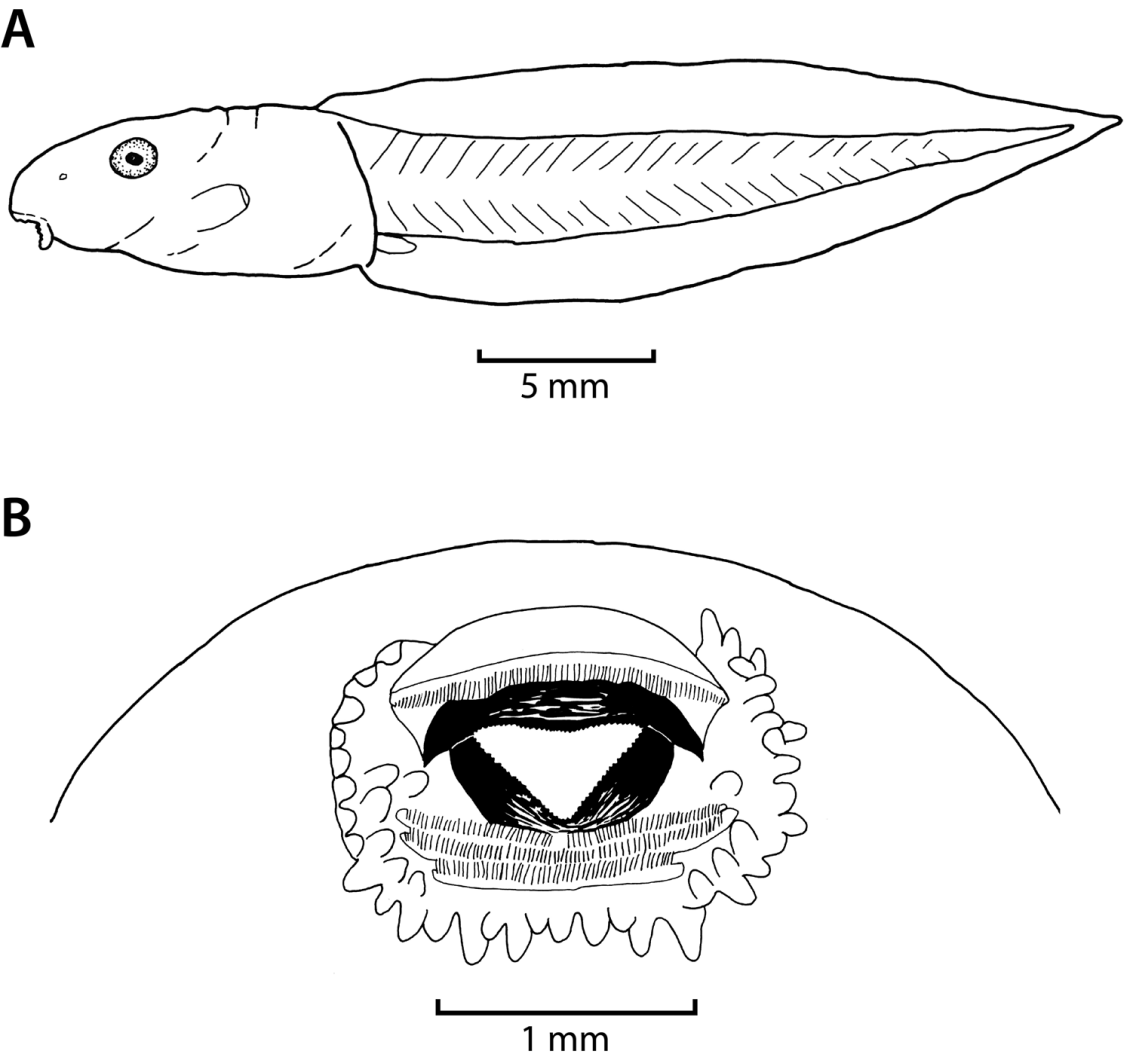
Tadpole of *Hyperolius jackie* (Stage 32, ZFMK 97193) in lateral view (**A**) and oral disc (**B**). Drawings by E. Lehr.

**Figure 12.**
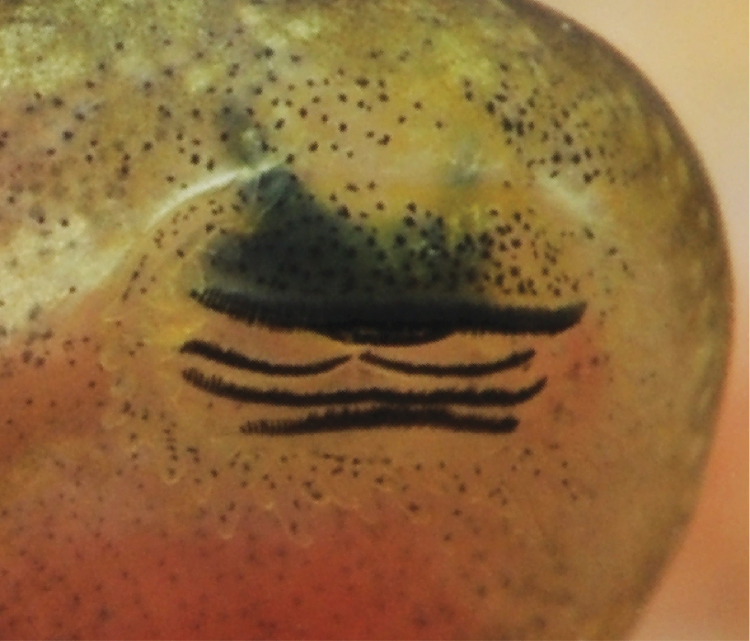
Oral disc in life of *Hyperolius jackie* (Stage 35, ZFMK 97194). Photo by M. Dehling.

**Figure 13.**
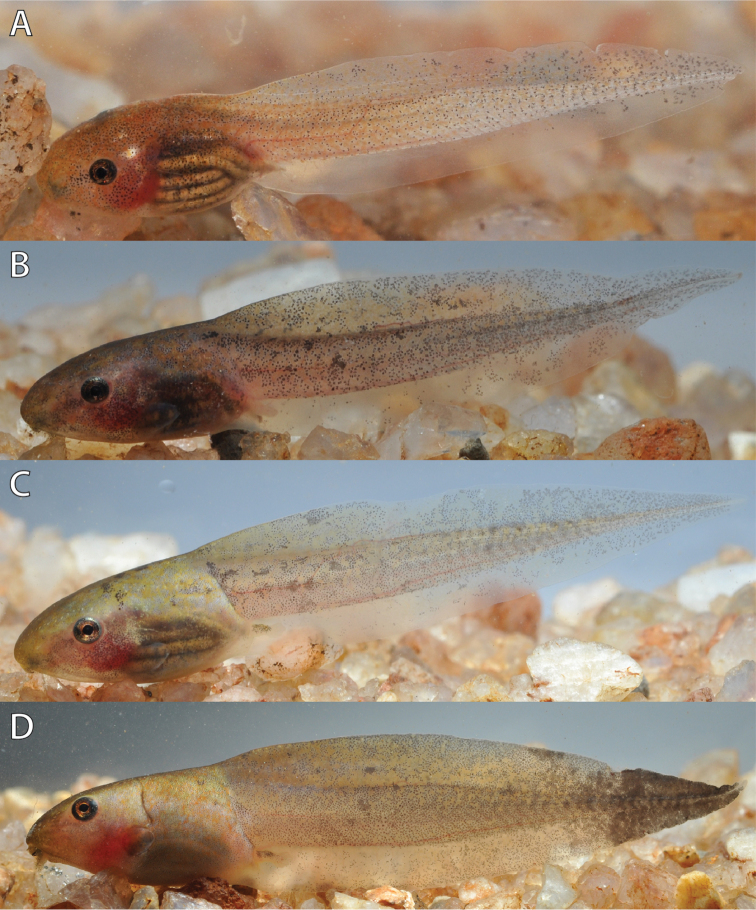
Color variation in life of *Hyperolius jackie* from Karamba (ZFMK 97194) at different Gosner stages. **A** Stage 25 **B** Stage 30 **C** Stage 34 **D** Stage 35. Photos by M. Dehling.

**Figure 14.**
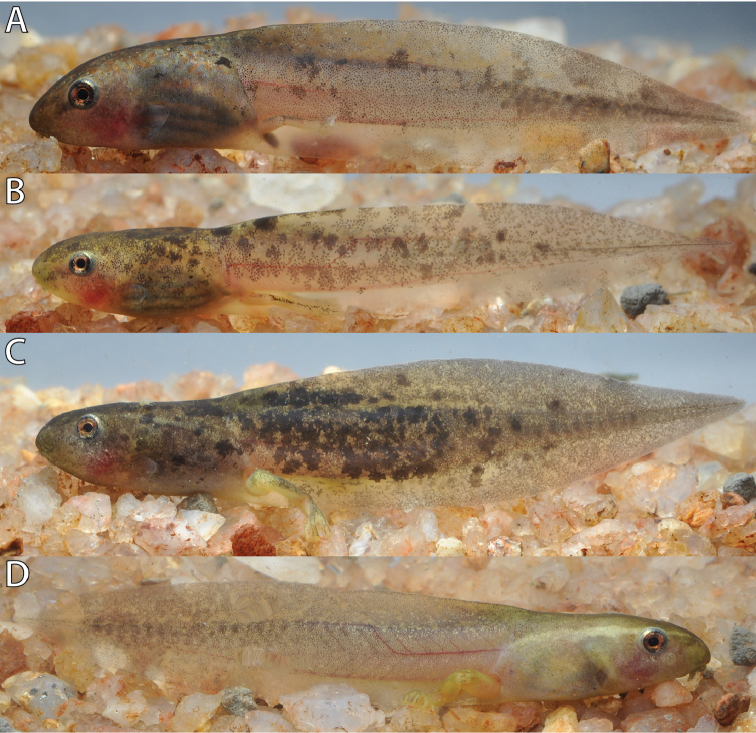
Color variation in life of *Hyperolius jackie* from Karamba (ZFMK 97194) at different Gosner stages. **A** Stage 36 **B** Stage 37 **C** Stage 40 **D** Stage 40. Photos by M. Dehling.

The following description is based on a Gosner Stage 32 individual from the Karamba swamp (Fig. 11, ZFMK 97193, from a series of 43 tadpoles, Gosner stages 25–41, ZFMK 97194, Figs 12–14). Exotrophous lentic benthic Type IV tadpole with the following measurements (mm): total length 31.5, body length 9.5, tail length 22.0, body width 5.2, body height 3.4, eye diameter 1.2, interorbital distance 4.8, internarial distance 3.0, distance–snout–naris 1.5, distance–naris–eye 1.6, spiracle length 1.9, spiracle width 0.6, distance–snout–spiracle 7.2, tail muscle height at its beginning 3.3, tail muscle height at tail mid-length 2.8, greatest tail height 6.8, oral disc width 1.6. In dorsal view the body is elongated and ovoid and is widest just posterior to the eye. The snout is rounded both in lateral and dorsal views. The interorbital distance is about three times the snout–naris–distance, and the internarial distance is 62.5% of the interorbital distance. The eyes are positioned laterally, directed dorsolaterally, and are slightly visible in ventral view. The external nares are ovoid and round (elongated horizontally), very small, and positioned laterally. They are nearly positioned in the middle between the eyes and snout (naris–eye–distance to snout–naris–distance 106.6%). In lateral view the body is highest at the mid-body length (approximately at the level of the spiracle opening). The body height is 36% of the body length, the body width is about half (55%) the length of the body, and the body height is 65% of the body width. The spiracle is single, sinistral, and attached to the body wall. Its shape is cylindrical and its length is 158% of the eye diameter. The spiracle opening is rounded, directed posteriorly, and located at mid-body with its upper margin reaching the level of the lower margin of the eye in lateral view. The length of the tail represents 70% of the total length. The tail is highest at about mid-tail and represents 31% of the tail length. The greatest tail height is 72% of the body length, and twice the body height. The dorsal fin does not extend onto the body. The dorsal fin is slightly higher than the ventral fin for about two thirds of the anterior tail length. The dorsal and ventral fins are of equal height for the posterior third of the tail. The tip of the tail is pointed and rounded. The height of the tail musculature at mid-body is slightly less than half (41%) of the maximum tail height. The vent tube is dextral, short, posteriorly directed, and linked to the tail musculature. The oral disc (Figs 11B, 12) is anteroventral, not emarginated, 31% of the body width, and bordered at its lateral and posterior margin by a row of short and round papillae. Few submarginal papillae are present laterally and below the third lower tooth row. The LTRF is 1/3(1) with a narrow median gap in P1. The first two tooth rows are about equal in length, occupying nearly the entire width of the oral disc, the third tooth row is slightly shorter, and the shortest is the most posterior one. Jaw sheaths are finely serrated. The upper jaw sheath is inversely U-shaped and the lower V-shaped and narrower.

The variation in external morphology of the larval series is limited to size (Table 4) and LTRF. Seven tadpoles differ from the above described LTRF: four had a LTRF of 1/3(1, 2), one of 1/3(1, 2, 3), one of 1/3, and one of 1/1.

**Table 4.** Measurements (mm) of 43 larvae of *Hyperolius jackie*. Mean followed by one standard deviation, and range in parentheses for sample sizes larger than 2.

*Hyperolius jackie*				
Stage	N	Total length	Body length	Tail length
**25**	2	16.0, 16.1	5.5, 5.6	10.5, 11.5
**26**	1	19.6	6.5	13.1
**28**	2	20.4	7.1	13.3
**30**	1	25.0	7.3	17.7
**31**	2	26.4, 31.5	8.5, 9.3	17.9, 22.2
**32**	2	30.8, 31.5	9.5, 9.7	21.1, 22.0
**34**	4	31.4–37.6 (33.6 ± 2.9)	10.0–11.4 (10.6 ± 0.6)	20.8–26.2 (23.0 ± 2.5)
**35**	6	25.8–35.3 (31.2 ± 3.5)	9.4–11.0 (10.0 ± 0.7)	16.1–24.7 (21.2 ± 3.1)
**36**	4	30.7–36.7 (32.8 ± 2.8)	9.1–11.7 (10.6 ± 1.1)	19.1–25.5 (22.2 ± 2.7)
**37**	4	35.1–42.2 (38.2 ± 3.1)	10.8–12.1 (11.5 ± 0.7)	24.2–30.2 (26.8 ± 2.5)
**38**	4	39.5–41.4 (40.1 ± 0.9)	11.7–12.2 (11.9 ± 0.2)	27.4–29.6 (28.2 ± 1.0)
**39**	1	43.5	12.7	30.8
**40**	4	38.9–44.2 (41.9 ± 2.2)	11.2–13.5 (12.3 ± 0.9)	27.7–32.0 (29.6 ± 1.9)
**41**	2	38.1, 43.7	10.9, 12.1	27.2, 31.6

In preservative the larvae are entirely pale grayish brown to tan. The body is darker dorsally compared to the translucent venter. The tail musculature is tan and the fins are translucent, both bearing dark gray melanophores in various degrees.

The coloration in life (Figs 13, 14) of the body was tan dorsally with minute brownish-orange and grayish-green spots and translucent whitish ventrally. The tail musculature was greenish tan and the fins were translucent tan with irregular dark marbling. Dark gray spots and flecks were scattered dorsally and laterally on the body, tail musculature and dorsal fin. The ventral fin has often fewer gray spots and flecks or is identical to the pattern of the dorsal fin (Figs 13A vs. 13C). Younger Gosner stages (e.g., Gosner Stage 25, Fig. 13A) are paler in overall coloration pattern compared to older Gosner stages (e.g., Gosner Stage 30, Fig. 13B). Individuals greatly differ in the amount of gray spots and flecks. Some have few gray spots and flecks scattered on the body and tail (Fig. 13C), whereas others have either numerous spots or flecks (Fig. 14C) or the tail tip can be nearly uniformly black (Fig. 13D). From Gosner stages 38 on, distinct tan or whitish yellow dorsolateral stripes are present on each side extending from the snout to the end of the body. The iris was brownish orange with a few dark gray reticulations.

### Differential diagnosis of bog pool tadpoles

In the Nyungwe National Park *Hyperolius castaneus* and *Hyperolius jackie* tadpoles may co-occur and share the same pool with *Leptopelis karissimbensis* or Leptopelis cf. kivuensis 2. The tadpole of *Leptopelis karissimbensis* has been described in detail before (Roelke et al. 2009), and that of the morphologically similar *Leptopelis kivuensis* briefly in Channing et al. (2012). At any stage the dark pigmented *Leptopelis* tadpoles are longer (e.g., total length for *Leptopelis karissimbensis* 51.4 mm at Gosner Stage 42 [Roelke et al. 2009], for Leptopelis cf. kivuensis 2 52.0 mm at Gonser Stage 39, for *Hyperolius castaneus* 31.0 mm at Gosner Stage 38, and *Hyperolius jackie* 43.7 mm at Gonser Stage 38) than *Hyperolius* tadpoles, mainly because of considerably longer tails. The tail fins are shorter in *Leptopelis karissimbensis* and *Leptopelis kivuensis*, and the LTRF in both species is 4(2–4)/3 vs. 1/3(1) in *Hyperolius castaneus* and *Hyperolius jackie*. Applying morphometrics on tadpoles of Gosner range 30–39, Gosner stage-adjusted body length and tail length and consequently total length differ significantly among species (ANCOVA, F_3,81_=21.0/67.9/62.3, P<<0.0001: *Hyperolius castaneus* (n = 56; BL = 10.6 mm; TAL = 19.2 mm; TL = 29.8 mm; least square means) < *Hyperolius jackie* (n = 34; BL = 11.5 mm; TAL = 24.7 mm; TL = 36.2 mm) < *Leptopelis karissimbensis* (n = 26; BL = 13.6 mm; TAL = 27.6 mm; TL = 41.2 mm) < Leptopelis cf. kivuensis 2 (n = 24; BL = 12.4 mm; TAL = 30.3 mm; TL = 42.6 mm). We have not recorded any differences in external morphology or coloration to distinguish the tadpoles of *Hyperolius castaneus* and *Hyperolius jackie*.

## Discussion

Eleven species of *Hyperolius* (*Hyperolius castaneus*, *Hyperolius cinnamomeoventris*, *Hyperolius discodactylus*, *Hyperolius frontalis* Laurent, 1950, *Hyperolius glandicolor* Peters, 1878, *Hyperolius kivuensis*, *Hyperolius jackie*, *Hyperolius lateralis*, *Hyperolius parallelus* Günther, 1858, *Hyperolius rwandae* Dehling, Sinsch, Rödel & Channing, 2013 in Channing et al. 2013, and *Hyperolius viridiflavus* Duméril & Bibron, 1841) are currently known to occur in Rwanda (Dehling 2012, unpubl. Data, Sinsch et al. 2011, 2012). Four of the Rwandan *Hyperolius* (*Hyperolius castaneus*, *Hyperolius discodactylys*, *Hyperolius frontalis*, and *Hyperolius jackie*) have been recorded in cloud forests of the Nyungwe National Park (Dehling 2012, unpubl. data), and three (*Hyperolius castaneus*, *Hyperolius cinnamomeoventris*, *Hyperolius glandicolor* [the latter recorded as *Hyperolius viridiflavus* by Roelke and Smith (2010), but species identification was corrected as *Hyperolius glandicolor* by Dehling, unpubl. data.]) in cloud forests of the Volcano National Park (Roelke and Smith 2010). The tadpoles of five species of Rwandan *Hyperolius* have been described: *Hyperolius castaneus* (this paper), *Hyperolius kivuensis* (Viertel et al. 2007), *Hyperolius jackie* (this paper), *Hyperolius lateralis* (Channing et al. 2012), and *Hyperolius viridiflavus* (Viertel et al. 2007), whereas the tadpole of *Hyperolius discodactylus* and *Hyperolius frontalis* will be described by Dehling and Sinsch in the near future. All five tadpoles share a LTRF of 1/3(1). At Gosner Stage 36 following total lengths (TL) have been reported (mean followed by range in parenthesis): *Hyperolius castaneus*: 31.9 ± 0.9 (30.8–33.0, n = 5); *Hyperolius jackie*: TL = 32.8 ± 2.8 (30.7–36.7, n = 4); *Hyperolius kivuensis*: TL = 34.9 (28.8–40.7, n = 14, Viertel et al. 2007); *Hyperolius lateralis*: unknown, 35 mm length given without stage assignment (Channing et al. 2012); *Hyperolius viridiflavus*: TL = 35.4 (30.0–39.6, n = 38, Viertel et al. 2007). Based on mean TL at this stage, the tadpole of *Hyperolius viridiflavus* is the largest, followed by *Hyperolius kivuensis*, *Hyperolius jackie*, and *Hyperolius castaneus* in descending TL, and unknown for *Hyperolius lateralis*. The external nares are positioned closer to the eyes than to the snout in *Hyperolius castaneus*, and positioned nearly in the middle between the eyes and snout in *Hyperolius jackie* and *Hyperolius lateralis*, whereas the external nares are more closely positioned to the snout than to the eyes in *Hyperolius kivuensis* and *Hyperolius viridiflavus*. Dorsal and ventral fins are about equal in height throughout their length in *Hyperolius castaneus*, whereas the dorsal fin is slightly higher than the ventral fin for about two thirds of the anterior tail length and of equal height for the posterior third, the upper tail fin is larger in height than the lower in *Hyperolius kivuensis* and *Hyperolius viridiflavus*, and condition unknown for *Hyperolius lateralis*. From Gosner stages 38 on, both *Hyperolius castaneus* and *Hyperolius jackie* tadpoles can be differentiated from the other three tadpoles in having distinct tan or whitish yellow dorsolateral stripes on each side extending from the snout to the end of the body. In summary, the observable differences in *Hyperolius* tadpoles are subtle as expected for cryptic species and some tadpoles (*Hyperolius lateralis*) need further investigations.

Viertel et al. (2007) were the first ones to describe oral disc and buccal cavity morphology in *Hyperolius* tadpoles and their value for taxonomy. Applying scanning electron microscopy, Viertel et al (2007) noted inter- and intraspecific differences in the types of labial teeth as well as interspecific differences in the buccal cavity. However, such methodology is relatively expensive and time intensive. Regarding external morphology, proportions, coloration and LTRF, *Hyperolius* tadpoles are very similar with only minor differences, which make species identifications unreliable, especially in areas with high species diversity, syntopic distributions or areas that have not been surveyed. This is the case for both *Hyperolius castaneus* and *Hyperolius jackie* larva, which only differ externally by their size (*Hyperolius jackie* larva are larger). We therefore consider DNA barcoding the most reliable method for identifications of larval *Hyperolius*, which was already noted by Viertel et al. (2007).

Dipteran predation on arboreal frog eggs in Africa was first described by Vonesh and Ross (2000) for four species of *Hyperolius* from Uganda. An infestation rate of 40% was recorded within the 1261 observed clutches of *Hyperolius lateralis*, *Hyperolius cinnamomeoventris*, *Hyperolius platyceps* (Boulenger, 1900), and *Hyperolius kivuensis*. Larvae of ephydrid and phorid flies feed on frog ova and cause high embryonic mortality, and the surviving tadpoles hatch at a smaller size (Vonesh and Ross 2000, Vonesh 2005). Our observation of an infestation of egg mass by larval dipterid flies in *Hyperolius castaneus* is to our knowledge the first record for this species.

With continuing fieldwork in Rwanda and other African countries, we are confident that the knowledge on reproduction, embryogenesis and species diversity of *Hyperolius* will increase.

## Appendix I

**Larval specimens examined**

*Hyperolius castaneus*: DR CONGO: North Kivu: Mt. Tshiaberimu, Virunga National Park (S00.12605, E29.43284, 2767 m a.s.l.) collected on 7 July 2008 by E. Greenbaum, C. Kusamba, W. M. Moninga and M. M. Aristote: UTEP 20620 (one tadpole, Gosner Stage 35), UTEP 20621 (one tadpole, Gosner Stage 37); RWANDA: Nyungwe National Park, from a natural pond forming part of the Uwasenkoko swamp (2.529°S, 29.354°E, 2379 m a.s.l.), collected on 22 and 26 March 2012 by J. M. Dehling & U. Sinsch: ZFMK 97191 (series of 51 tadpoles, Gosner stages 25–38), ZFMK 97190 (single tadpole selected from ZFMK 97191, Gosner Stage 29); Nyungwe National Park, from a natural pond at Karamba (2.479°S, 29.112°E, 1936 m a.s.l.), collected on 24 March 2012 by J. M. Dehling & U. Sinsch: ZFMK 97192 (series of 5 tadpoles, Gosner stages 34–41); UGANDA: Muchuya Swamp, Stream (S01.25543, E29.79689, 2200 m a.s.l.) collected on 25 May 2014 by E. Greenbaum, D. F. Hughes and M. Behangana: UTEP 21179 (one tadpole, Gosner Stage 37).

*Hyperolius jackie*: RWANDA: Nyungwe National Park, from a natural pond at Karamba (2.479°S, 29.112°E, 1936 m a.s.l.), collected on 24 March 2012 by J. M. Dehling & U. Sinsch: ZFMK 97194 (series of 42 tadpoles, Gosner stages 25–41), ZFMK 97193 (single tadpole selected from ZFMK 97194, Gosner Stage 32).

*Leptopelis karissimbensis*: RWANDA: Nyungwe National Park, from a natural pond forming part of the Uwasenkoko swamp (2.529°S, 29.354°E, 2379 m a.s.l.), collected on 22 and 26 March 2012 by J. M. Dehling & U. Sinsch: ZFMK 97188 (series of 26 tadpoles, Gosner stages 25–39).

Leptopelis cf. kivuensis 2: RWANDA: Nyungwe National Park, from a natural pond at Karamba (2.479°S, 29.112°E, 1936 m a.s.l.), collected on 24 March 2012 by J. M. Dehling & U. Sinsch: ZFMK 97189 (series of 24 tadpoles, Gosner stages 28–39).
